# The health system burden of chronic disease care: an estimation of provider costs of selected chronic diseases in Uganda

**DOI:** 10.1111/tmi.12487

**Published:** 2015-03-06

**Authors:** Stella Nalukwago Settumba, Sedona Sweeney, Janet Seeley, Samuel Biraro, Gerald Mutungi, Paula Munderi, Heiner Grosskurth, Anna Vassall

**Affiliations:** ^1^ Medical Research Council/Uganda Virus Research InstituteEntebbeUganda; ^2^ London School of Hygiene and Tropical MedicineLondonUK; ^3^ Department of Non Communicable DiseasesMinistry of HealthKampalaUganda; ^4^ Mwanza Intervention Trials UnitMwanzaTanzania

**Keywords:** non‐communicable diseases, human immunodeficiency virus, cost, low‐ and middle‐income countries, Uganda, health systems, Maladies non transmissibles, VIH, coût, pays à revenus faibles et intermédiaires, Ouganda, systèmes de santé

## Abstract

**Objective:**

To explore the chronic disease services in Uganda: their level of utilisation, the total service costs and unit costs per visit.

**Methods:**

Full financial and economic cost data were collected from 12 facilities in two districts, from the provider's perspective. A combination of ingredients‐based and step‐down allocation costing approaches was used. The diseases under study were diabetes, hypertension, chronic obstructive pulmonary disease (COPD), epilepsy and HIV infection. Data were collected through a review of facility records, direct observation and structured interviews with health workers.

**Results:**

Provision of chronic care services was concentrated at higher‐level facilities. Excluding drugs, the total costs for NCD care fell below 2% of total facility costs. Unit costs per visit varied widely, both across different levels of the health system, and between facilities of the same level. This variability was driven by differences in clinical and drug prescribing practices.

**Conclusion:**

Most patients reported directly to higher‐level facilities, bypassing nearby peripheral facilities. NCD services in Uganda are underfunded particularly at peripheral facilities. There is a need to estimate the budget impact of improving NCD care and to standardise treatment guidelines.

## Abstract (fr)

**Objectif:**

Investiguer sur les services des maladies chroniques en Ouganda: leur niveau d'utilisation, les coûts totaux des services et les coûts unitaires par visite.

**Méthodes:**

Les données complètes sur les coûts financiers et économiques ont été collectées dans 12 établissements de deux districts, selon la perspective du prestataire. Une combinaison d'approches de calcul des coûts basé sur les ingrédients et une répartition par étape a été utilisée. Les maladies étudiées étaient le diabète, l'hypertension, la bronchopathie pulmonaire chronique obstructive (BPCO), l’épilepsie et l'infection VIH. Les données ont été collectées au moyen d'un examen des dossiers de l’établissement, l'observation directe et des entretiens structurés avec les agents de la santé.

**Résultats:**

Les prestations de services de soins chroniques étaient concentrées dans les établissements de niveau supérieur. A l'exclusion des médicaments, les coûts totaux pour les soins des maladies non transmissibles (MNT) sont tombés en dessous de 2% des coûts totaux de l’établissement. Les coûts unitaires par visite variaient considérablement, à la fois entre les différents niveaux du système de santé et entre les établissements de même niveau. Cette variabilité était dirigée par les différences dans les pratiques cliniques et de prescriptions des médicaments.

**Conclusion:**

La plupart des patients se présentaient directement dans les établissements de niveau supérieur, contournant ainsi les établissements périphériques de proximité. Les services des MNT en Ouganda sont sous financés, notamment dans les établissements périphériques. Il est nécessaire d'estimer l'impact budgétaire de l'amélioration des soins des MNT et de normaliser les directives de traitement.

## Abstract (es)

**Objetivo:**

Explorar los servicios de atención a enfermedades crónicas en Uganda: su nivel de uso, los costes totales del servicio y costes unitarios por visita.

**Métodos:**

Se recogieron datos financieros totales y costes económicos de 12 centros en dos distritos, desde la perspectiva del proveedor. La asignación de costes se realizó combinando la estrategia basada en ingredientes y la escalonada (*step‐down*). Las enfermedades estudiadas fueron diabetes, hipertensión, enfermedad pulmonar obstructiva crónica (EPOC), epilepsia e infección por VIH. Los datos se recogieron mediante una revisión de las historias clínicas, observación directa y entrevistas estructuradas con los trabajadores sanitarios.

**Resultados:**

La entrega de servicios de atención a crónicos estaba concentrada en centros de mayor nivel. Excluyendo los fármacos, los costes totales para la atención a enfermedades no infecciosas (ENI) cayeron por debajo del 2% del total de los costes del centro. Los costes unitarios por visita variaban ampliamente, tanto a diferentes niveles del sistema sanitario como entre centros sanitarios de un mismo nivel. Esta variabilidad se debía a las diferencias en las prácticas clínicas y de prescripción de fármacos.

**Conclusión:**

La mayoría de los pacientes se dirigían directamente a los centros de mayor nivel, independientemente de tener otros centros periféricos más cerca. La financiación de los servicios de atención a ENI en Uganda es insuficiente, particularmente en el caso de centros periféricos. Es necesario calcular el impacto del presupuesto en la mejora de la atención a ENI, y estandarizar las guías de tratamiento.

## Introduction

The prevalence of non‐communicable disease (NCD) has been rising in low‐ and middle‐income countries (LMIC) over the past years and is likely to increase further 1, 2. A survey performed in 23 LMIC found that NCDs (mainly diabetes, cardiovascular diseases, cancers and chronic respiratory diseases) were responsible for 50% of the total disease burden in 2005 3. According to the Global Burden of Disease Study 2010, although communicable, maternal, neonatal and nutritional diseases rank highest in causes of premature mortality in eastern sub‐Saharan Africa, the percentage of deaths due to these health problems generally declined, while the percentage due to NCDs substantially increased between 1990 and 2010 4. Many LMICs are therefore now facing a ‘double burden’ due to the increasing levels of NCDs and continuing occurrence of infectious diseases including HIV infection, which is now a chronic disease (CD) 5. The WHO Kampala Declaration and Agenda for Global Action of 2008 (‘health workers for all and all for health workers’) 6 emphasises the dilemma of this double burden, and the pressure it places on health systems and the limited resources available to fund them^7^.

However, despite this growing pressure, there remains little understanding about how chronic care services are and should be provided in LMIC, particularly at the peripheral health system level. While there has been progress in disease‐specific chronic care programs targeted at people with human immunodeficiency virus (HIV) infection, NCDs have not yet seen structured care programmes in LMIC 8. This lack of a programmatic approach exists despite the fact that NCDs and HIV infection require similar responses from the health system: a regular long‐term follow‐up of patients and an uninterrupted provision of medicines. Furthermore, many countries are concerned about healthcare expenditures associated with NCDs and how best to provide services within their scarce resources to meet the growing added demand for chronic services 9. In particular, policymakers need country‐specific financial information on the costs of care for NCDs, to plan, budget and assess the cost‐effectiveness of any expansion in NCD service delivery. However, most available evidence on the current economic burden on the health system for providing health services for these diseases is from high‐income settings and these are not transferable to LMICs 10; therefore, appropriate data on the costs of NCDs in LMICs remain extremely scarce.

The objective of this study was to present data on current service utilisation and the total and unit (average per service) costs observed for chronic care from twelve health facilities of different levels in Uganda aiming to provide useful information for the country's emerging NCD control programme. As Uganda has a well‐developed HIV treatment programme for many years, and HIV is now seen as a chronic condition, we included data on HIV care to help estimate the costs of comprehensive service from chronic conditions. The study was conducted as part of various preparations for a randomised controlled trial to investigate the feasibility and cost‐effectiveness of interventions to strengthen the health systems’ response to chronic diseases, to be conducted in collaboration between local research institutions and Ministries of Health in Uganda and Tanzania.

## Methods

### Study setting

The study was carried out in 2012 with the aim of establishing the availability, utilisation and costs of NCD and HIV care services, at 12 purposefully selected health facilities in Mpigi and Wakiso districts in southern Uganda, areas with both rural and urban populations. The diseases considered were diabetes, hypertension, epilepsy, chronic obstructive pulmonary disease (COPD) and asthma, and HIV infection.

The health facility types selected included one general hospital, one health centre level IV (HC IV), seven health centres level III (HCs III) and three health centres level II (HCs II). Two HCs III and one HC II were private not for profit (PNFP), while the others were public government‐funded HCs. Every Parish is supposed to have an HC II serving a few thousand people. This is led by an enrolled nurse and will treat common illnesses and may have antenatal services. Every subcounty, with a population of around ten thousand people, should have an HC III led by a clinical officer. HCs III have an outpatients department (OPD), a maternity unit, a functioning laboratory and may have a small inpatients department. An HC IV serves a county with a population of around 100 000. In addition to the services available at HCs III, it commonly has an inpatients department with male, female and paediatric wards and a theatre for emergency operations. HCs IV are led by medical officers.

### Data collection

All cost data were collected from March 2012 to January 2013. We retrospectively collected full financial and economic costs for the calendar year 2011, as the last full year of audited financial data available at the time. We adopted the provider perspective, considering all costs regardless of who paid or funded them. Costs were obtained for all outpatient services within the health facility and then apportioned and analysed with a focus on the diseases under study. We assessed COPD together with asthma because in clinical practice, they were rarely distinguished. A combined ‘bottom‐up’ ingredients approach for disease‐specific costs and step‐down cost allocation method for overhead costs was used in the costing 11.

Data on quantities or resource use and prices for each CD were collected through a review of facility records, interviews and observation of service utilisation and entered into structured cost data sheets. In preparation, to understand the service delivery system for CDs, semi‐structured interviews were carried out among key health facility personnel. The interviews included questions on the services available and how they are provided and organised, human resource availability, facility organogram and staff time allocated to different departments, supervision structures, drugs and other supplies distributed to each facility and the occurrence of stock‐outs, referral systems, and the organisation of specialised clinics for diabetes, epilepsy and HIV infection that were held at some facilities. Prior to interviews, observations and data collection, written informed consent was obtained from heads of facilities and other health workers using an information sheet and consent form.

Costs were classified as capital and recurrent costs. For full details on the sources of both quantity and price data used, see Appendix 1. Capital costs included building space, equipment and staff training. All capital costs were annualised at a discount rate of 3% 12. Building costs were measured by mapping the facility and assigning the building space to different service areas using staff interviews. Where several services took place in one space, the space costs were allocated based on the type, amount and time used to provide each service. The price of building space was estimated using market prices for rent for similar buildings in the same areas. Equipment costs were assigned to the different cost centres depending on what the equipment was used for and the percentage of patients for each service that used the equipment. Equipment prices were sourced from 2011 supplier catalogues. Staff training costs were assigned depending on the type of service the training was for. The cost of training per day was sourced from interviews with training organisations.

Recurrent costs included drugs and diagnostics, transport, staff time, supplies and utilities. Staff costs included salaries and allowances such as housing, lunch and uniform. Staff costs were allocated to the different disease costs using the amount of time staff spent working on those conditions obtained through staff interviews and observation. Apart from HIV treatment, which has strictly followed prescription standards, there were no records found for a complete list of drugs used by condition for the entire year. To estimate annual drug costs, we collected detailed drug utilisation data for the previous month at the time the facility was visited to understand typical drug prescription patterns in each health facility. Data on drug prescriptions were obtained from the OPD register, clinic registers for diabetes and epilepsy and quarterly reports for HIV infection. Drug prices were obtained from 2011 supplier catalogues. Drugs were only costed if they were actually prescribed and provided at a facility; drugs that were prescribed but then purchased by patients on their own costs were not included.

Transport costs were obtained from facility expenditure records and vehicle and motorcycle log books. General supplies and utilities costs were obtained from monthly bills and facility expenditure records and allocated to overheads. Laboratory costs were excluded in the analysis due to unavailability of data in many facilities and the fact that many facilities were not equipped to carry out relevant laboratory tests. It should be noted that this is an omission, and in some instances due to stock‐out of testing reagents, the government health facilities would independently organise to purchase reagents and continue providing diagnostic services; however, patients had to pay out of pocket. This was particularly the case for diabetes.

### Data analysis

To assign overhead and support costs to cost centres, we attached the costs to specific departments; these included support services (pharmacy and laboratory), overhead (administration, maintenance and laundry and cleaning) and medical care services (the diseases being studied). We assigned a portion of support and overhead service costs to final diagnosis‐related cost centres using step‐down accounting methods 13 based on staff numbers for administrative costs or use of physical space for maintenance, laundry and cleaning.

To estimate unit costs per visit for each disease from total costs, data for the total monthly outpatient visits were obtained from the monthly and quarterly Health Management Information System (HMIS) reports. This included an assessment of the number of patients with CD covered each month and the diagnosis and treatment they received.

Although chronic care costs are often estimated per person‐year of treatment 14, 15, 16, we were not able to estimate yearly or episode costs because with the exception of HIV care at larger facilities there was little continuity of care; and medical records were not available for a cohort of patients that was followed up continuously for long periods.

Data were analysed in Microsoft Excel. All costs were collected in Ugandan shillings and then presented in 2012 United States dollars (USD). The average 2012 exchange rate of 2582.57 Uganda shillings to 1 USD was used 17.

## Results

### Service organisation and availability

Table 1 describes the services provided at each level of the health system in 2011. Generally, CD services were more available at higher‐level health facilities. Inpatient services were mainly available at the hospital and HC IV. Some speciality drugs (e.g. some diabetes and antihypertensive drugs) were only distributed to HCs IV and hospitals; these drugs were therefore only accessible to patients at higher levels of the health system. Drug prescription patterns varied between facilities, even at the same level. Apart from HIV infection for which drug refills were provided monthly, the periods for which a patient was given drugs without review lasted 3–4 months at some facilities and for much shorter periods, a few days or weeks, at others. Drug refills are free at all government facilities and are paid for by the patient at private facilities. Many times government facilities reported drugs stock‐outs especially for diabetes. In such instances, written prescriptions were given to patients who then had to buy the drugs from a private pharmacy or drug shop. As these are patient costs, they were not included in the analysis. While the hospital and HC IV had the minimum number of the staff required according to staffing norms, only 1 of 7 HCs III and 1 of 3 HCs II had all the staff allocated to them. Many facilities did not have functional laboratories and where these existed, they were mainly used for malaria tests; and equipment needed for other diagnostic tests was unavailable or not functioning. Some facilities had weekly or monthly clinics for certain diseases. These were run by staff with specialised training in the management of certain conditions.

**Table 1:** Description of services provided at the different levels of health facilities in 2011/2012

Level	Health facility type	Highest cadre of staff available	Service availability														
			Diabetes			Hypertension		COPD/Asthma		Epilepsy			HIV infection				
			Special clinic	Laboratory diagnosis and monitoring	Drug refills	Diagnosis and monitoring (BP machine)	Drug refills	Clinical diagnosis	Drug refills	Clinical diagnosis	Special clinic	Drug refills	Special clinic	CD4 count monitoring	Pre‐ART	ART	Diagnosis (HCT)
Parish	HC II Govt	Enrolled nurse				x	x	xx	xx	x					x		
	HC II PNFP	Enrolled nurse			x	xx	xx	xx	xx								x
Sub‐County	HC III Govt	Clinical officer				x	xx	xx	xx	xx	x	xx	x	x	xx	x	x
	HC III PNFP	Clinical officer		xx	xx	xx	xx	xx	xx	x		x					xx
County	HC IV Govt	Medical doctor		xx	xx	xx	xx	xx	xx	xx		xx	xx	xx	xx	xx	xx
District	Hospital	Specialised medical doctor	xx	xx	xx	xx	xx	xx	xx	xx	xx	xx	xx		xx	xx	xx

x, some facilities; xx, all facilities.

In the absence of a regular clinic, conditions are managed in the general outpatients department.

Govt, Government owned public facility; PNFP, Private not for profit facility; HCT, HIV counselling and testing.

Full diabetes services were only available at the hospital and at PNFP facilities. Services included laboratory diagnosis and monitoring of blood or urine glucose using glucometer sticks or urine dipstick tests, regular monitoring of blood pressure and drug refills. The hospital ran a specialised diabetes clinic twice a week in which lab diagnosis, monitoring and drug refills were offered, and hypertension was treated as a comorbidity. Commonly used diabetic drugs were metformin and glibenclamide. Insulin was sometimes available although frequent stock‐outs were reported. The HC IV diagnosed diabetes and then referred patients to the hospital for treatment. Diagnosis was usually performed using a urine glucose dipstick test. Health workers reported frequent and long‐lasting stock‐outs of dipsticks and in such instances patients had to pay for them after improvisations were made by the facilities to independently purchase them. Smaller facilities did not provide diabetes care but occasionally provided symptomatic treatment if needed. They usually referred patients to higher facilities. Sometimes patients were given a prescription for diabetes drugs by the clinicians and asked to buy the drugs at private pharmacies and drug shops.

Hypertension services were available at all levels except at 1 of the 2 government HCs II and were provided by the OPD. Services available included diagnosis and monitoring using a blood pressure machine and drug refills; however, blood pressure machines used for diagnosis were not available at 2 of 3 HCs II and 1 of 7 HCs III. In these cases, patients were sometimes asked to have their blood pressure taken at a private facility and results returned. The hypertensive drugs commonly prescribed at lower‐level facilities were propranolol, furosemide and nifedipine, while higher‐level facilities additionally prescribed methyldopa, losartan, bendroflumethiazide, atenolol and captopril.

Care for patients with COPD/asthma comprised clinical diagnosis and drug refills and was available at all levels of the healthcare system and provided through general OPD services. Officers in charge of health facilities mentioned that COPD is usually not distinguished from asthma and is reported as ‘asthma’ in OPD registers at all facilities including the hospital. COPD is also not included on the HMIS report forms. We therefore collected data on ‘asthma’ in adults; however, this included both cases of asthma and COPD. COPD/asthma services available were clinical diagnosis and drug refills and both conditions were generally treated in OPDs. The most commonly prescribed drugs were prednisolone, salbutamol and aminophylline.

Epilepsy services were based on clinical diagnosis and included monitoring and drug refills. The hospital ran a weekly specialised epilepsy clinic under the mental health unit, staffed with full‐time psychiatry nurses, and usually monthly inpatient services were also available. The HC IV provided care for epileptic patients as part of general OPD services but also had a resident psychiatric nurse. 2 of 7 HCs III operated regular monthly epilepsy clinics run by a visiting psychiatry specialist from the mental health department of the district health office, while others did this as part of general OPD care and usually only provided drug refills. None of the HCs II included in this study offered services for epileptic patients. Common drugs prescribed for epilepsy were phenobarbital, phenytoin and carbamazepine.

HIV services were provided as HIV care without antiretroviral therapy (ART) for patients on cotrimoxazole only, or HIV care with ART provision. HIV care was available at all HCs III, HC IV and the hospital, while ART services were only available at the hospital, HC IV and 3 of 7 HCs III. At HCs II, HIV infection was suspected based on clinical diagnosis and referrals made. 2 of 3 HCs II gave patients cotrimoxazole refills.

Table 2 shows the mean number of outpatient visits per year by each facility level. Generally, higher‐level health facilities received more patients with chronic disease than lower‐level facilities. HIV‐related services registered the highest number of visits even at lower‐level health facilities. The largest number of visits for HIV care, 10 398, was recorded at the HC IV, which included visits received at outreach clinics, especially in communities where HIV services were not offered at lower‐level facilities.

**Table 2:** Annual number of OPD visits and total annual cost by disease and facility level (2012)

	Type of service	Health facility	Mean annual visits		Mean total service cost (USD 2012)		% Cost for each mean cost type			
			Mean	Range	Mean	Range	Drugs (%)	Staff (%)	Capital (%)	Other (%)
NCD Services	COPD/Asthma	Hospital	511		847		2	40	52	6
		HC IV	63		128		4	52	42	2
		HC III	35	12–66	74.98	44–114	25	55	17	3
		HC II	21	13–29	42	29–59	26	51	20	3
	Diabetes	Hospital	3632		8002		5	54	36	5
		HC IV	6		22		15	38	45	2
		HC III	34	0–62	123	0–459	25	46	27	2
		HC II	N/P	N/P	N/P	N/P	N/P	N/P	N/P	N/P
	Epilepsy	Hospital	1368		6698		42	16	38	4
		HC IV	284		510		21	30	47	2
		HC III	27	0–62	147	0–519	20	52	25	3
		HC II	N/P	N/P	N/P	N/P	N/P	N/P	N/P	N/P
	Hypertension	Hospital	1219		5668		53	14	30	3
		HC IV	245		730		34	36	29	1
		HC III	117	11–349	351	44–1218	27	45	25	3
		HC II	31	0–32	68	0–117	12	42	43	3
HIV Services	HCT	Hospital	5296		13124		–	5	56	39
		HC IV	4983		10017		–	17	39	44
		HC III	746	381–1892	2893	563–6287	–	48	26	26
		HC II	7	0–7	8	0–17	–	62	12	26
	HIV Care/ART	Hospital	4529		79345		64	13	22	1
		HC IV	10398		164279		49	7	42	2
		HC III	3673	3325–4020	32378	31799–32959	41	30	27	2
		HC II	N/P	N/P	N/P	N/P	N/P	N/P	N/P	N/P
	HIV Care only	HC III	1093	63–1081	11753	1003–22512	4	61	31	4
		HC II	31	24–36	68	59–85	30	50	18	2

Hospital (*n* = 1), HC IV (*n* = 1), HC III (*n* = 7), HC II (*n* = 3).

HCT, HIV counselling and testing; N/P, Service not provided;

HIV care only are patients not yet initiated on ART.

Other includes transport, utilities and general facility supplies including sundries and stationary.

Patient visits due to hypertension were recorded at all levels of health facilities, particularly at the hospital. Almost all visits due to diabetes occurred at the hospital and very few at other facility levels. The few diabetes patient visits at the HC III level were recorded at private facilities. For epilepsy, 1368 patient visits were registered at the hospital's regular clinic and 284 visits at the HC IV. However, the numbers of epilepsy visits recorded at HCs III was very low, only 27, despite the fact that these facilities offered monthly epilepsy clinics.

### Total costs

Table 2 also shows the mean total costs by disease for each level of health facility. The disease service area that had the highest cost burden was HIV, with NCDs only incurring a very small fraction of the costs. At the hospital, HC IV and HCs III, the annual total costs for HIV services are higher than the combined total cost of the NCD services. Among the NCD services, COPD/asthma had the least total cost burden. Across the different levels of facilities, for all diseases, the hospital and HC IV had very high total costs compared to lower‐level facilities, with the hospital bearing almost all the cost burden for diabetes.

The balance across inputs varied. While costs for HIV care were mainly driven by drug costs, accounting for 64% and 49% of total costs at the hospital and HC IV, respectively, those for other NCDs were primarily driven by capital and staff costs.

**Figure 1:**
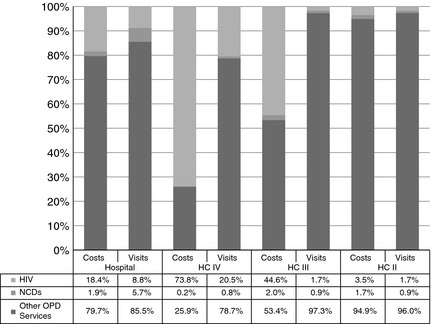
Total outpatient HIV and NCD costs and visits as a percentage of facility outpatient costs and visits.

Figure 1 shows the percentage of total facility costs, excluding drug costs, spent on HIV care, NCD care and all other OPD services and the corresponding percentage of visits made by patients for each disease, at the different levels of health services. We excluded drug costs to provide an indication on the health system burden (in terms of fixed costs such as capital and human resources) of different disease groups relative to service utilisation. Costs for HIV care and other CDs together amounted to approximately 20%, 75%, 47% and 5% of total non‐drug‐related OPD costs at hospital, HC IV, HC III and HC II levels, respectively. The majority of this was for HIV care. The total costs for NCD care were below 2% of total OPD costs at all health facilities. At HC IV and the hospital, the burden of NCDs in terms of visits is much higher than their percentage cost allocation. At the HC III and II, where little or no NCD services are provided, the cost allocation is a little higher than the burden.

### Unit costs

Table 3 presents unit costs per visit for each facility. Unit costs for HIV services, particularly HIV care with ART, were much higher than those for NCD services. Unit costs for HIV/ART services ranged from USD 8.91 to USD 17.52, and were driven primarily by higher drug and capital costs. Unit costs for NCD services were all under USD 7 per visit; and there were no substantial differences between them.

**Table 3:** Non‐Communicable disease and HIV Services unit costs per visit (2012 USD)

	Hospital (*n* = 1)	HC IV (*n* = 1)	HC III (*n* = 7)		HC II (*n* = 3)	
	Unit cost per visit	Unit cost per visit	Unit cost per visit		Unit cost per visit	
			Mean	Range	Mean	Range
NCD services						
COPD/Asthma	1.66	2.04	2.36	1.49–3.70	2.10	1.47–2.77
Salaries	0.67	1.05	1.31	0.74–2.43	1.17	0.71–1.95
Drugs	0.04	0.08	0.61	0.27–1.34	0.44	0.02–0.69
Capital	0.86	0.87	0.37	0.19–0.77	0.43	0.15–0.68
Other	0.09	0.04	0.06	0.02–0.17	0.06	0.003–0.11
Diabetes	2.20	3.63	6.55	1.44–11.76		N/P
Salaries	1.18	1.40	3.34	0.99–7.15		N/P
Drugs	0.11	0.52	1.20	0.37–3.24		N/P
Capital	0.80	1.65	1.85	0.43–3.94		N/P
Other	0.11	0.05	0.07	0.02–0.3		N/P
Epilepsy	4.90	1.79	6.91	2.33–20.75		N/P
Salaries	0.80	0.54	3.82	0.74–9.90		N/P
Drugs	2.06	0.38	1.17	0.33–1.75		N/P
Capital	1.87	0.84	1.73	0.25–8.32		N/P
Other	0.17	0.04	0.16	0.02–0.79		N/P
Hypertension	4.65	2.98	3.50	1.59–6.59	3.29	2.69–3.89
Salaries	0.67	1.05	1.32	0.74–2.47	1.40	0.84–1.95
Drugs	2.49	1.02	0.48	0.99–1.27	0.38	0.32–0.44
Capital	1.37	0.87	1.64	0.50–5.46	1.43	1.35–1.51
Other	0.12	0.04	0.06	0.07–0.11	0.06	0.02–0.17
HIV services						
HCT	2.48	2.01	4.11	1.48–7.69	2.41	2.41
Salaries	0.12	0.35	1.99	0.42–4.94	1.10	1.10
Drugs	–	–	–	–	–	–
Capital	1.39	0.78	1.07	0.16–1.84	0.39	0.39
Other	0.97	0.88	1.05	0.90–1.34	0.31	0.92
HIV Care only			3.65	1.03–6.15	2.30	1.64–3.53
Salaries			2.29	0.53–3.57	1.17	0.71–1.95
Drugs			0.56	0.39–0.78	0.65	0.39–0.78
Capital			0.73	0.09–1.70	0.43	0.15–0.69
Other			0.07	0.00‐ 0.13	0.06	0.00–0.11
HIV Care and ART	17.52	15.80	8.91	7.91–9.91		
Salaries	2.31	1.08	2.76	1.19–4.34		
Drugs	11.19	7.71	3.32	0.51–6.13		
Capital	3.81	6.75	2.62	0.46–4.79		
Other	0.21	0.25	0.2	0.13–0.27		

N/P: Service not provided at this level of health facility.

HCT, HIV counselling and testing.Other includes transport, utilities and general facility supplies including sundries and stationary.

Across the different levels of facilities, mean unit costs for COPD/asthma were similar, approximately USD 2 per visit at all facilities, while those for diabetes and epilepsy were up to three times higher at HCs III than at the HC IV and hospital. The highest unit cost for hypertension was also observed at HCs III. Some HCs III were able to provide services at the same costs as HC IV and hospitals. The pattern in cost variability across facility levels was primarily driven by salaries, which were approximately USD 3 per visit at HC III but only around USD 1 at HCs IV and the hospital. It was also driven by different levels of drug costs.

The unit costs for PNFP facilities were higher than those of public government facilities. This was seen in the unit costs of diabetes, which was USD 2.2 at the hospital, USD 3.63 at the HC IV and USD 6.55 per visit at HC III, where only private facilities have diabetes services. Private facilities also had higher unit costs for salaries, capital and drugs, which were often paid for by the patients.

## Discussion

Our study found that the provision of chronic care services in our area was patchy and primarily concentrated at higher‐level facilities rather than at the periphery, suggesting that the population in the mostly rural catchment areas of these health facilities remain underserved with respect to these diseases. HIV and hypertension services were more widely available than services for other CDs such as epilepsy and diabetes. The low coverage at the periphery may be due to the fact that patients with chronic disease may delay seeking care and therefore may often need more specialised services. However, it is more likely reflective of the fact that many lower facilities are not yet able to diagnose and provide appropriate services for NCDs. While the problem of a rising disease burden from NCDs has been recognised in Uganda and elsewhere in sub‐Saharan Africa 1, 7, our study suggests that health services at the periphery do not yet reflect this transition. The costs of NCDs as a proportion of overall health expenditure were negligible, even at higher levels of care.

Where chronic services were provided across all levels of the health system (e.g. for COPD/asthma), there were still differences in resource allocation across levels, with larger facilities having higher levels of total costs and resources, but also receiving proportionally more specialised drugs and equipment. For specialised healthcare services, this may be inevitable, but our findings suggest that standard services for NCDs that could be provided routinely at smaller health facilities require substantially more resources to cope with the growing burden.

While differences in resource allocation may be determined by the types and quality of services on offer, the unit costs of care for some disease were lower at larger facilities, which may be explained by the fact that much higher numbers of patients are seen suggesting possible economies of scale. This in turn suggests that an increase in allocation of resources to peripheral health facilities, should it result in an increased level of provision of services, may lead to a similar effect – and reduce the variation between facilities observed – particularly if ‘fixed’ costs like staff can be used more efficiently.

The high variability in costs between facilities of the same level was in part driven by differences in drug costs. We observed the greatest variability in drug cost per visit for epilepsy, hypertension and COPD/asthma, and yet, the same supplier with a standard price was used. This variability occurred because prescriptions differed substantially between facilities even for the same drug and for similar conditions. The introduction of standard treatment algorithms and prescription guidelines would probably be cost saving. In addition, achieving a more reliable supply of drugs is likely to increase adherence and reduce clinic workloads and may also have a positive knock‐on effect on other costs and overall efficiency 8.

We also observed that private facilities paid higher salaries to their staff, procured more expensive drugs if deemed necessary and had better equipment. However, private facilities charge patients for their services, while in Uganda healthcare provision in the public sector is in principle free. In this study, we did not collect data on health care from the user perspective; and an all‐inclusive comparison of public and PNFP health services was not possible therefore.

Because our study did not assess the costs of CDs to individual patients, we also do not know the cost burden of these diseases on households. However, it is likely that the current underinvestment in the health system with respect to NCDs may result in overall economic loss to households and even the society, particularly if persons with NCDs need to travel significant distances to receive care, seek care with delay or not at all. This is aggravated by the fact that the time and transport costs to access appropriate care may simply be out of reach for the poor or those living on subsistence farming who have no sufficient cash income.

Uganda is currently making major efforts to improve chronic care through its emerging NCD control programme. The cost data collected in this study will contribute to planning and to costing the scale‐up of NCD services. The health system investment required to expand NCD services could be minimised if attention is focused on how to organise these services cost‐effectively. The successes of HIV and tuberculosis programmes in Africa are encouraging and could provide good examples for NCD care, keeping in mind that a successful integration of services would allow cost‐effective use of healthcare resources 8. This has been documented in other studies. An example is given by a study from Cameroon where the integration of hypertension and diabetes services was found to be feasible and affordable to patients 18.

Our study had several limitations. As continuous NCD services are still the exception, data are scarce and our ability to observe services in real time was limited. This may lead to an underestimation of cost, as we may have been unable to identify and capture all resource use. The use of routine data also presented a substantial challenge, although great care was taken to validate data through other methods such as staff interviews. The cost data collected excluded laboratory costs, as these were not available for many of the facilities. We were also unlikely to capture the overall burden on the health system from NCDs. Some of the complications of NCDs may be experienced in other departments, if not recognised as being due to NCDs and this again may have biased our results to underestimate total NCD expenditures. Nevertheless, our study remains one of the few available with empirical cost estimates for NCD services in a real world context, and we hope is an important source of cost data for those seeking to scale up NCDs in LMICs.

## Conclusion

Our research suggests that NCD services are still chronically underfunded, particularly at peripheral health facilities in Uganda, as is the case in many other African countries that are facing a similar rapidly growing disease burden from NCDs. Our study emphasises the need to evaluate current services and estimate the budget impact of improving or introducing NCD care at lower‐level health facilities. There is a particular need for standardisation of treatment guidelines especially drug prescriptions and for making drugs more widely available and to strengthen service models that are integrated both within and between facilities. We expect that the study will contribute to the efforts of the emerging public NCD control programme in Uganda and elsewhere.

## Appendix 1

### Sources of resource costs

**Table:**

Resource		Source of information
Capital	Staff training	Key staff interviews, health facility staff training records, training organisations.
	Buildings	Building measurements, market rental prices.
	Equipment	Ingredients approach, market replacement costs from 2011 supplier catalogues.
Recurrent	Drugs	Ingredients approach, drug prescription data from facility records, 2011 drug price lists from supplier catalogues.
	Transport	Vehicle and motorcycle log books, petty cash expenditure records.
	Staff time	Staff interviews, work duty rosters, staff observations, human resource records, salaries and allowances obtained from the government salary structure for the financial year 2010/2011.
	Supplies	Facility records, supplier catalogues.
	Utilities	Monthly bills for electricity, water, telephone services (where applicable) and security, security staff salaries and allowances, facility expenditure records.
